# Getting off to a good start

**DOI:** 10.7554/eLife.88396

**Published:** 2023-05-11

**Authors:** Igor Martianov, Irwin Davidson

**Affiliations:** 1 Institut de Génétique et de Biologie Moléculaire et Cellulaire Strasbourg France

**Keywords:** embryonic stem cells, transcription, degron, genomics, gene expression, Mouse

## Abstract

RNA polymerase II transcription can efficiently occur when mouse embryonic stem cells lack TBP and TBP-like proteins, confirming that this initiation factor may not be as essential as once thought.

**Related research article** Kwan JZJ, Nguyen TF, Uzozie AC, Budzynski MA, Cui J, Lee JMC, Van Petegem F, Lange PF, Teves SS. 2023. RNA polymerase II transcription independent of TBP in murine embryonic stem cells. *eLife* **12**:e83810. doi: 10.7554/eLife.83810.

Transcription requires a large number of proteins to come together in a precise order to read a particular segment of the genome and copy it into RNA. Switching on a protein-coding gene, for instance, involves a set of highly evolutionary conserved transcription factors to assemble at the site where transcription begins (Roeder, 2019). Together, they form a preinitiation complex that bends and opens up the DNA so that the enzyme RNA polymerase II can position itself to transcribe the gene (Figure 1).

**Figure 1.**
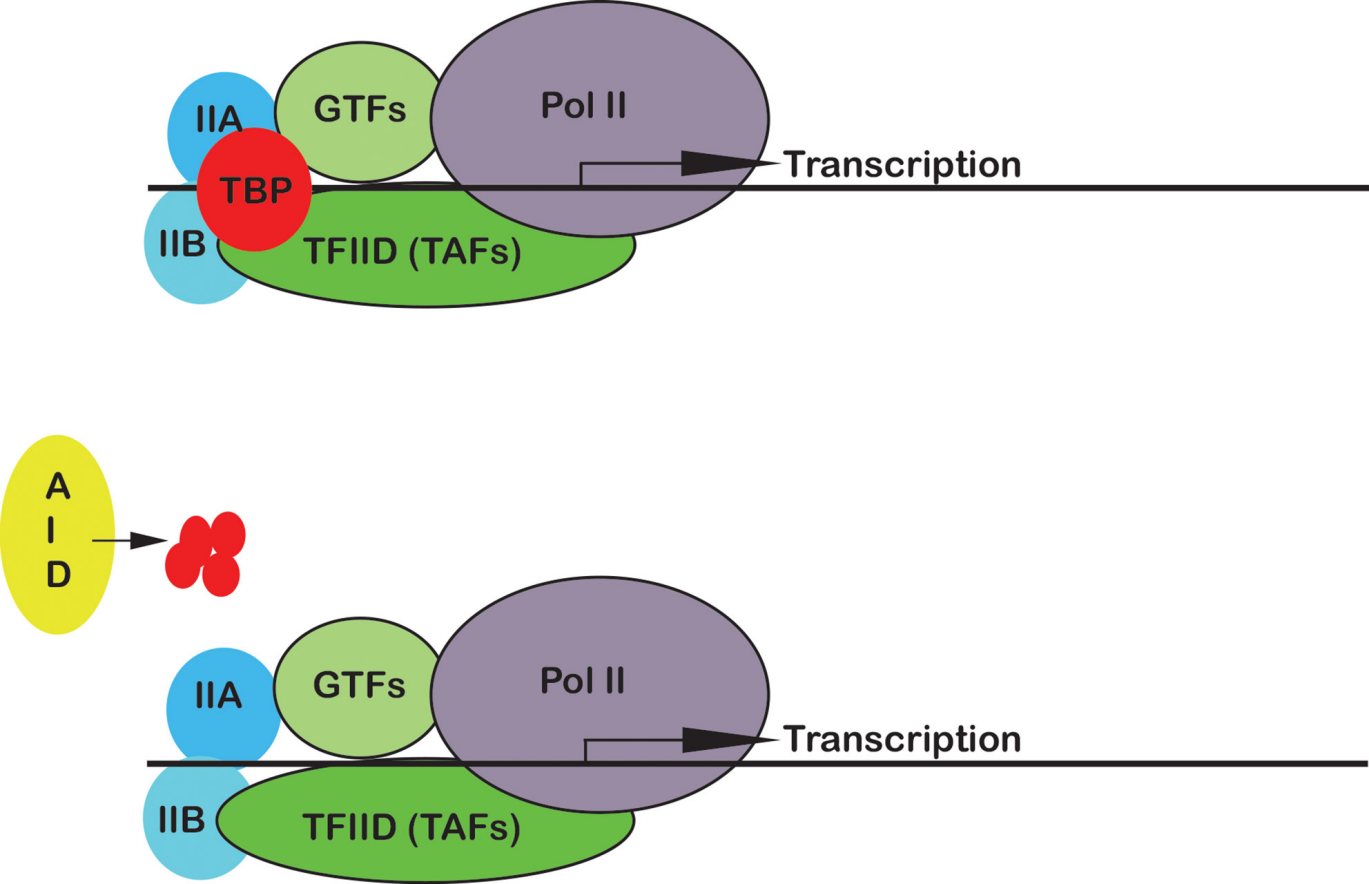
Transcription can take place without TBP in mouse embryonic stem cells. Before a gene can be transcribed (top panel), the preinitiation complex must assemble around the site where transcription begins (indicated by an arrow). The complex is made up of a number of components, including the enzyme RNA polymerase II (Pol II; purple), TBP (red), TFIIA (IIA; dark blue), TFIIB (IIB; light blue), TFIID (dark green), and other general transcription factors (GTFs; light green). Kwan, Nguyen et al. found that breaking down the protein TBP using an auxin-inducible degradation system (AID; lime green) did not impact the formation of the preinitiation complex or stop RNA polymerase II from transcribing genes (bottom panel). Abbreviations: TBP (TATA-element binding protein); TFIIA (transcription factor II A); TFIIA (transcription factor II B); TFIID (transcription factor II D).

The first protein to bind to DNA is usually TBP (short for TATA-box binding protein), which then promotes the remaining components of the preinitiation complex to attach to the transcription start site. While TBP can initiate this process on its own, it normally carries out this role as one of the many subunits of transcription factor TFIID, which also contains 13 TBP-associated factors (TAFs). TBP became a central focus of the transcription field when it was discovered that all three nuclear RNA polymerases found in eukaryotes (RNA polymerase II but also RNA polymerase I and III, which transcribe non-protein coding RNA molecules) require TBP, elevating it to the status of ‘universal transcription factor’ (Hernandez, 1993).

However, the genomes of multi-cellular eukaryotes also encode TBP-like proteins, known as TBP-related factor 2 (TRF2) and TBP-related factor 3 (TRF3), which may replace TBP to ensure RNA polymerase II transcription in specific developmental processes. So far, the role of TRF2 and TRF3 has been respectively restricted to male and female germ cells in mammals, and to the transcription of developmentally regulated genes in other animals (Martianov et al., 2001; Zhang et al., 2001; Martianov et al., 2016; Gazdag et al., 2009). Yet, overall, the exact function of these TBP-like proteins remains poorly understood.

Subsequent studies then revealed that RNA polymerase II – but not RNA polymerase I and III – could in fact transcribe DNA without TBP or similar proteins (Wieczorek et al., 1998; Martianov et al., 2002). However, exactly how this TBP-independent transcription occurs has remained unclear. Now, in eLife, Sheila Teves and colleagues – including James Kwan and Thomas Nguyen as joint first authors – report that RNA polymerase II can transcribe genes in mouse embryonic stem cells when TBP is degraded and both TRF2 and TRF3 are absent (Kwan et al., 2023).

The team (who are based at the University of British Columbia and BC Children’s Hospital Research Institute) used an auxin-based system which utilizes the cell’s own processes to rapidly and synchronously degrade TBP proteins. The amount of TBP and RNA polymerase II bound to the promoter sites where transcription begins was then quantified together with the level of newly generated RNA molecules.

Kwan, Nguyen et al. found that although the level of TBP was reduced by almost 90%, this did not compromise the integrity of TFIID. However, the recruitment of another TFIID subunit called TAF4, as well as the transcription factor TFIIA, was reduced. The level of RNA polymerase II bound to promoter sites and involved in transcription (measured by nascent RNA production) remained essentially the same, whereas RNA polymerase III transcription was strongly inhibited. Importantly, TBP degradation in embryonic stem cells where the gene for TRF2 was inactivated also did not reduce RNA polymerase II transcription, suggesting that TRF2 does not compensate for the loss of TBP. In these conditions, RNA polymerase II transcription occurs without TBP and TBP-like proteins (as TRF3 is not expressed in embryonic stem cells and therefore cannot play a compensating role).

Further experiments revealed that TBP depletion did not affect the activation of previously unexpressed genes induced by heat shock or retinoic acid treatment. This suggests that preinitiation complexes do not need TBP to switch on previously inactive genes or to maintain the transcription of the ones which are already turned on.

This work represents one of the most comprehensive mechanistic studies on how TBP depletion affects RNA polymerase II transcription in mammalian cells, but many questions remain. The findings of Kwan, Nguyen et al. suggest that there is no linear relationship between TBP levels and RNA polymerase II transcription; however, it is possible that the residual 10% of TBP left behind after degradation could be playing some yet undefined role. If so, it would be clearly at odds with established ideas of the role of TBP in transcription initiation, requiring a serious revision of current models. Furthermore, it remains to be seen if the DNA bending normally induced by TBP still happens in its absence, and if so how this occurs. It is possible that TBP loss is compensated for by contacts made by the other subunits of TFIID with the DNA and other proteins of the preinitiation complex, as Kwan, Nguyen et al. showed that the integrity and recruitment of these components were not strongly affected by TBP depletion.

If TBP is not required to form new preinitiation complexes or reinitiate transcription, then what is its role? One possibility is that mouse embryonic stem cells do not need TBP for transcription as they have very open chromatin environments, making it easier for RNA polymerase enzymes to access and bind to DNA – a situation that would not apply to most other cell types. Another suggested role for TBP is in mitotic book marking, as it remains bound to DNA during mitosis so certain genes can be quickly reactivated after division (Teves et al., 2018; Xing et al., 2008; Wang et al., 2021). Overall, the findings of Kwan, Nguyen et al. consolidate and enrich observations and ideas that were first formulated more than 20 years ago. Hopefully, the next decades will finally bring clarity about how the preinitiation complex can assemble without needing TBP.
